# Treatment expectations and goals among patients with chronic myeloid leukemia in Germany: a patient-centered perspective

**DOI:** 10.1038/s41375-025-02826-w

**Published:** 2025-12-19

**Authors:** Philipp Ernst, Cera Lohse, Michael Lauseker, Jan Geißler, Philipp le Coutre, Tim H. Brümmendorf, Susanne Saußele, Andreas Burchert, Georg-Nikolaus Franke, Paul La Rosée, Annamaria Brioli, Thomas Schenk, Christian Fabisch, Thomas Ernst, Guido Mehlkop, Andreas Hochhaus

**Affiliations:** 1 Klinik für Innere Medizin II, Universitätsklinikum Jena, Comprehensive Cancer Center Central Germany, Campus Jena, Jena, Germany; 2 Institute for Planetary Health Behaviour, Universität Erfurt, Erfurt, Germany; 3 Institut für Medizinische Informationsverarbeitung, Biometrie und Epidemiologie (IBE), Ludwig-Maximilians-Universität, München, Germany; 4 LeukaNET e.V., Riemerling, München, Germany; 5 Klinik für Hämatologie, Onkologie und Tumorimmunologie, Charité—Universitätsmedizin Berlin, Berlin, Germany; 6 Klinik für Onkologie, Hämatologie und Stammzelltransplantation, Universitätsklinikum RWTH Aachen, Center for Integrated Oncology (CIO) Aachen Bonn Cologne Düsseldorf (CIO ABCD), Aachen, Germany; 7 III. Medizinische Klinik, Universitätsmedizin Mannheim, Universität Heidelberg, Mannheim, Germany; 8 Klinik für Hämatologie, Onkologie und Immunologie, Universitätsklinikum Marburg, Marburg, Germany; 9 Klinik und Poliklinik für Hämatologie, Zelltherapie, Hämostaseologie und Infektiologie, Universitätsklinikum Leipzig, Comprehensive Cancer Center Central Germany, Campus Leipzig, Leipzig, Germany; 10 Klinik für Innere Medizin II, Schwarzwald-Baar Klinikum, Villingen-Schwenningen, Germany; 11 Klinik für Hämatologie, Hämostaseologie, Onkologie und Stammzelltransplantation, Medizinische Hochschule Hannover, Hannover, Germany

**Keywords:** Quality of life, Chronic myeloid leukaemia

## Abstract

Chronic myeloid leukemia (CML) is a chronic condition with excellent long-term survival under tyrosine kinase inhibitor (TKI) therapy. However, patient priorities regarding treatment goals and quality of life remain insufficiently understood. We conducted a nationwide online survey among German CML patients in collaboration with the German CML Alliance and patient organizations to assess treatment goals at diagnosis, during therapy, and in the treatment-free remission phase. The questionnaire included validated measures of treatment satisfaction, fear of progression, and quality-of-life priorities, supplemented by newly developed items. Between November 2024 and February 2025, 582 patients (median age 56 years, 48.8% female) completed the survey. Overall TKI tolerability was rated positively (median 4/5), particularly in first-line therapy and among patients with shorter disease duration, whereas long-term survivors reported more daily-life limitations. Younger patients ( < 45 years) emphasized fertility, sexuality, and work-related concerns, while older patients prioritized tolerability, independence, and mental health. Treatment history shaped expectations: those with discontinuation experience were more willing to accept adverse effects for the prospect of remission. Overall, patient priorities diverged between achieving deep molecular response and maintaining optimal tolerability. Integrating patient-reported preferences into shared decision-making may enhance satisfaction, adherence, and long-term outcomes of patients with CML.

## Introduction

Expectations of cancer patients regarding treatment goals and needs are influenced by a multitude of factors that encompass demographic, socioeconomic, psychosocial, and clinical domains [1]. While younger patients often focus on preservation of reproductive capacity, older patients are mainly concerned with quality of life and coping with co-morbidities [2]. Treatment preferences and information needs differ between men and women and are also dependent on ethnic and cultural background [3]. Individual beliefs and goals, including attitudes towards quality and duration of life, as well as ensuring that patients are well informed and their preferences are respected, play a crucial role in treatment decisions [4]. In addition to the specific cancer diagnosis and the level of progression, individual health conditions determine the urgency and aggressiveness of treatment as well as the feasibility and choice of therapeutic interventions.

Chronic myeloid leukemia (CML) is a myeloproliferative disorder that is diagnosed in chronic phase in more than 97% of cases and has a very good prognosis with an 8-year survival rate of up to 95% on tyrosine kinase inhibitors (TKIs) [5]. Although the median age of onset is 66 years, about 17% of patients are younger than 45 years of age [6]. Young adults and adolescents may have concerns regarding fertility and parenting. While TKIs in general do not have a negative impact on male fertility, they pose maternal and fetal risks during pregnancy, which affect treatment decisions and quality of life [7]. The response to the initial therapy significantly influences the therapy objectives, as achieving and maintaining a deep molecular response (DMR) are primary goals, especially when considering subsequent treatment-free remission (TFR) [8, 9]. For the selection of appropriate first-line therapy, the patients existing health conditions and comedications are key determinants in the physicians decision-making. This is particularly relevant for baseline cardiovascular risk factors and diseases such as obesity, diabetes mellitus, and arterial hypertension, which are present in approximately 30% of CML patients and necessitate adjustment to the side-effect profiles of the available TKIs [10]. Patient adherence to prescribed therapies is crucial for achieving optimal results. Factors such as life circumstances, multiple medications and personal payment obligations can influence treatment choice and adherence and vary between countries and health care systems [11]. Resistance and intolerance to ATP-competitive TKIs has been described in 25% of patients and often necessitate a change in treatment [12]. There is a lack of data on patient priorities for disease management, long-term quality of life concerns and the role of patients in treatment decision-making. To reduce the likelihood of unnecessary treatment changes, improve quality of life and optimize goal achievement, the perspectives of patients and clinicians must be aligned. Based on the favorable long-term survival data under TKI therapy, new drugs should be evaluated according to alternative criteria such as tolerability, i.e. the absence of chronic, low-grade side effects, safety during long-term use, quality of life, speed of response, the possibility of interrupting therapy and costs [13]. Doctors and pharmaceutical companies are not in a position to make a definitive prioritization of CML patients’ goals from an external perspective. A systematic analysis of patients’ wishes and goals is therefore necessary. In cooperation between the German CML Alliance and the patient organizations Patvocates (Riemerling, Germany) and Leukaemie-Online (LeukaNET e.V., Riemerling, Germany), a systematic survey of German CML patients was therefore conducted at the time of diagnosis, during treatment and after discontinuation of therapy in order to obtain reliable information on the changed therapy goals from the patients’ perspective.

## Material and methods

### Conception of the questionnaire

A quantitative questionnaire was developed to assess multiple domains of CML treatment goals and satisfaction. Its design followed a dual approach combining adapted items from validated instruments with newly developed questions. Items were adapted from the *Cancer Therapy Satisfaction Questionnaire* [14, 15], the *Fear of Progression Questionnaire* [16], and surveys assessing quality-of-life priorities [17] and treatment side effects [18]. Socio-demographic items were derived from the *German Health Update (GEDA)* survey [19] and the *Health Information National Trends Survey (HINTS) Germany* [20, 21]. Study-specific questions were developed by a multidisciplinary panel comprising oncologists, health communications researchers, and a patient representative to capture aspects such as individual tolerability, knowledge and perceptions of TKI therapy, as well as personal treatment goals and preferences. Prior to the start of the nationwide survey, a total of 50 CML patients at Jena University Hospital received a pretest version of the questionnaire, which they were asked to evaluate for comprehensibility, feasibility and scope. The questionnaire was adapted accordingly.

### Preparation and conduction of the survey

The questionnaire was prepared using LimeSurvey version 6.4 for online access and completion. No personal data was processed. The survey was conducted nationwide in the period between November 1st, 2024 and February 28th, 2025. Access to the online questionnaire was granted through a QR code distributed by treating physicians or made available via Leukaemie-online.de, as part of a joint initiative between the German CML Alliance and the patient organization Patvocates.

### Informed consent and ethics committee approval

Patients were informed in writing of the voluntary nature, anonymity and purpose of the survey and the type of data storage and use was explained. All methods were performed in accordance with the relevant guidelines and regulations. The Ethics Committee of the Jena University Hospital reviewed and approved the survey (2024-3588-Bef).

### Statistical analysis

For group comparisons with ordinal-scaled data (5- or 7-point rankings), Mann–Whitney U tests were applied. For large sample sizes, the U statistic was standardized to its asymptotic normal distribution, and the corresponding *Z* value was reported. Effect sizes were expressed as *r* = *Z / √N* (rank-biserial correlation), interpreted as small (≈ 0.1), medium (≈ 0.3), or large ( ≥ 0.5) [22]. For associations between categorical variables, χ² tests were used, and Cramer’s *V* was reported as a sample size–independent measure of association, with the same interpretive thresholds. Statistical analysis was performed using GraphPad software (Version 8.2.0, Boston, MA, USA). The minimum target sample size was estimated a priori to ensure 95% confidence intervals for key proportions with a half-width of ≤5%, while maintaining 80% power to detect medium subgroup effects (r ≈ 0.3, α = 0.05). This corresponded to a minimum sample size of 500 participants.

## Results

### Demographics, cohort definition

A total of 582 adult CML patients completed the survey. The median age of participants was 56 years (range, 18–88 years), and gender distribution was nearly balanced (48.8% female patients). Forty (6.9%) participants were within their first year after diagnosis, 38 (6.5%) in their second year, and 377 (64.8%) had been diagnosed more than five years prior to the survey. Regarding treatment history, 221 (38.3%) of patients reported having received three or more lines of TKI therapy (Table 1). At the time of data collection, 142 (24.2%) patients had already discontinued TKI treatment. 60.1% of respondents reported being employed, including 39.0% in full-time positions, while 30.6% were retired (Supplementary Table 1). Knowledge and perceptions of CML differed between cohorts, with age, disease duration, and treatment experience being key determinants (Supplementary Table 2).

**Table 1:** Sociodemographic and clinical characteristics of patients with chronic myeloid leukemia (CML) who participated in the survey (*n* = 582).

		Time since diagnosis (months)			
	Total	1-12	13-24	25-60	>60
	*n* = 582	*n* = 40	*n* = 38	*n* = 127	*n* = 377
Median age, years (range)	56 (18–88)	57 (24–73)	56 (27–75)	54 (19–85)	56 (18–88)
<45 years, n (%)	135 (23)	13 (37)	8 (21)	45 (36)	71 (19)
45–65 years, n (%)	326 (56)	23 (63)	22 (58)	60 (47)	218 (58)
>65 years, n (%)	121 (21)	4 (10)	8 (21)	22 (17)	88 (23)
Median illness period, month (range)	85 (4–419)				
Sex, n (%)					
Female	284 (49)	24 (60)	21 (55)	65 (51)	175 (46)
Male	298 (51)	16 (40)	17 (45)	62 (49)	202 (54)
Current treatment, n (%)					
Imatinib	71 (12)	3 (8)	3 (8)	15 (12)	51 (14)
Nilotinib	95 (16)	7 (18)	16 (42)	22 (17)	49 (13)
Dasatinib	145 (25)	23 (58)	6 (16)	41 (32)	75 (20)
Bosutinib	27 (5)	1 (3)	3 (8)	8 (6)	15 (4)
Ponatinib	8 (1)	2 (5)	0 (0)	1 (1)	5 (1)
Asciminib	89 (15)	1 (3)	8 (21)	26 (20)	54 (14)
PEG-IFNα2a	2 (0)	1 (3)	0 (0)	0 (0)	1 (0)
Allogeneic SCT	6 (1)	2 (5)	0 (0)	2 (2)	2 (1)
Experimental therapies	5 (1)	0 (0)	2 (5)	2 (2)	1 (0)
None	134 (23)	0 (0)	0 (0)	10 (8)	124 (33)
Treatment line, n (%)					
1st line	128 (22)	23 (57)	17 (45)	38 (30)	52 (14)
2nd line	233 (40)	13 (33)	13 (34)	47 (37)	160 (42)
≥3rd line	221 (38)	4 (10)	8 (21)	42 (33)	165 (44)
After allogeneic SCT	12 (2)	1 (2)	0 (0)	1 (1)	10 (3)
Discontinuation, n (%)					
On TKI (never withdrawn)	297 (51)	37 (93)	35 (92)	109 (86)	116 (31)
On TKI (after withdrawal)	143 (25)	2 (5)	0 (0)	6 (5)	136 (36)
TKI withdrawn	142 (24)	1 (2)	3 (8)	12 (9)	125 (33)

Data are stratified by time since diagnosis. Shown are age distribution, median illness duration, sex, current treatment, treatment line, and treatment discontinuation status. Percentages are given in parentheses.

*PEG-IFN* pegylated Interferon, *SCT* stem cell transplantation, *TKI* tyrosine kinase inhibitor.

### Prior treatment tolerability

**Fig. 1:**
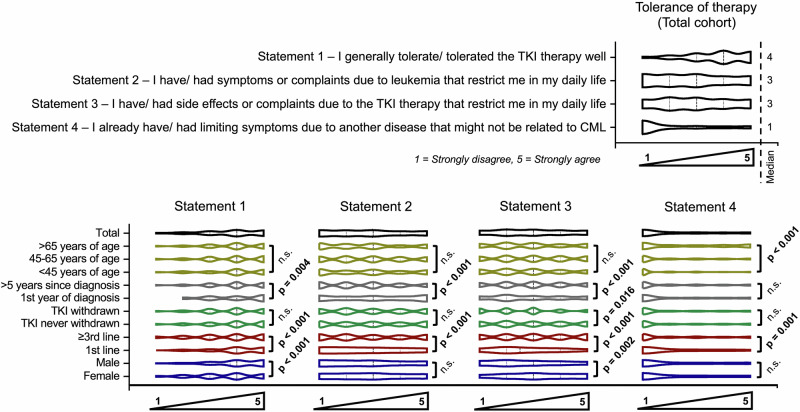
Patient-reported outcomes on tolerance of tyrosine kinase inhibitor (TKI) therapy in chronic myeloid leukemia (CML). Violin plots illustrate responses to four statements on therapy tolerance and symptom burden (1 = strongly disagree, 5 = strongly agree) in the total cohort and subgroups stratified by age, time since diagnosis, treatment line, TKI discontinuation status, and sex. *p* values indicate significant group differences; n.s. not significant.

Data on treatment tolerability were collected in order to stratify treatment goals. Overall tolerability of TKI therapy was rated positively, with a median score of 4 on a 5-point scale. Higher tolerability ratings were more commonly reported among male participants (Z = –5.5, *p* < 0.001, r = 0.23), individuals receiving first-line therapy (Z = –9.7, p < 0.001, r = 0.40), and those with shorter disease duration (Z = –2.9, *p* = 0.004, r = 0.18) (Fig. 1, Supplementary Table 2). Patients in later lines of therapy and with disease duration exceeding five years more frequently reported treatment-related limitations in daily life (Z = -18.1, *p* < 0.001, r = 0.85 and Z = –10.5, *p* < 0.001, r = 0.51, respectively). Symptom burden also varied by disease phase and age: patients closer to diagnosis reported more leukemia-associated symptoms (Z = –6.0, *p* < 0.001, r = 0.29), while participants aged over 65 years reported a higher prevalence of non-leukemia-related symptoms compared to younger patients under 45 years (Z = –3.8, *p* < 0.001, r = 0.16) (Fig. 1).

### Expectations concerning treatment

**Fig. 2:**
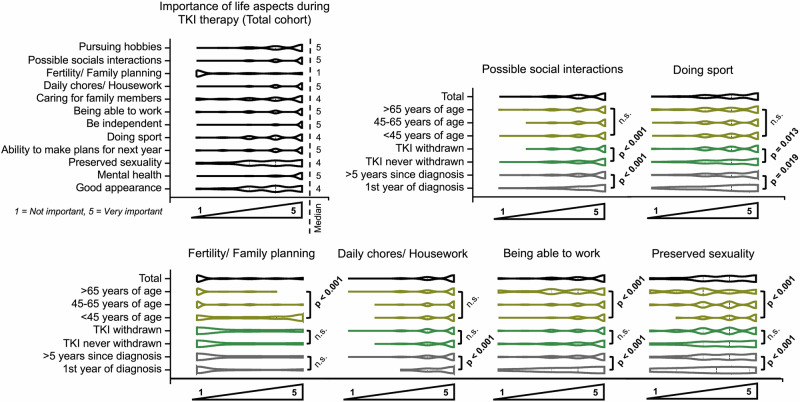
Importance of life aspects during tyrosine kinase inhibitor (TKI) therapy in patients with chronic myeloid leukemia (CML). Violin plots show ratings (1 = not important, 5 = very important) for different life domains in the total cohort and subgroups stratified by age, sex, time since diagnosis, treatment line, and TKI discontinuation status. Significant group differences are indicated (*p* values); n.s. not significant.

**Fig. 3:**
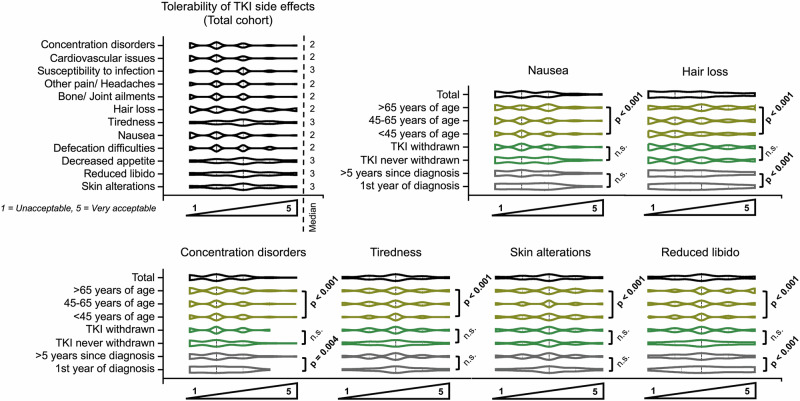
Tolerability of tyrosine kinase inhibitor (TKI) side effects in patients with chronic myeloid leukemia (CML). Violin plots show Likert scale ratings (1 = unacceptable, 5 = very acceptable) for different side effects, stratified by age, time since diagnosis, and TKI discontinuation status. Significant differences are indicated by *p* values; n.s. not significant.

**Fig. 4:**
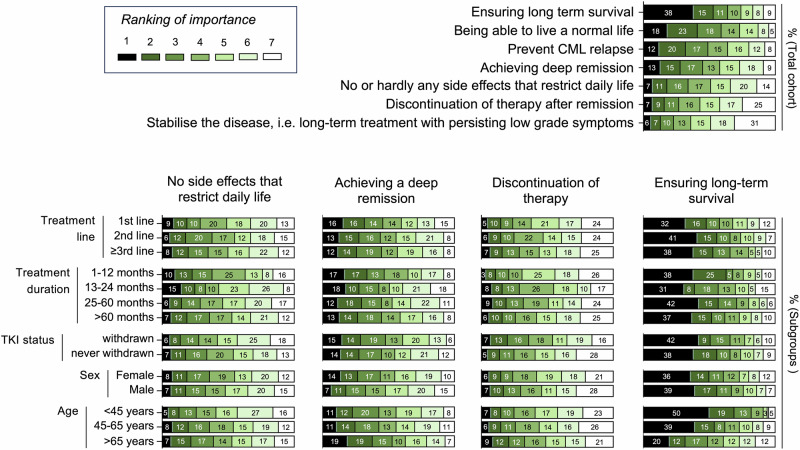
Preferences of treatment goals reported by patients with chronic myeloid leukemia (CML). Heatmaps display the ranking of importance (1 = highest, 7 = lowest) for different treatment goals in the total cohort (top right) and stratified by treatment line, time since diagnosis, TKI discontinuation status, sex, and age (bottom). Percentages indicate the distribution of rankings within each subgroup.

**Fig. 5:**
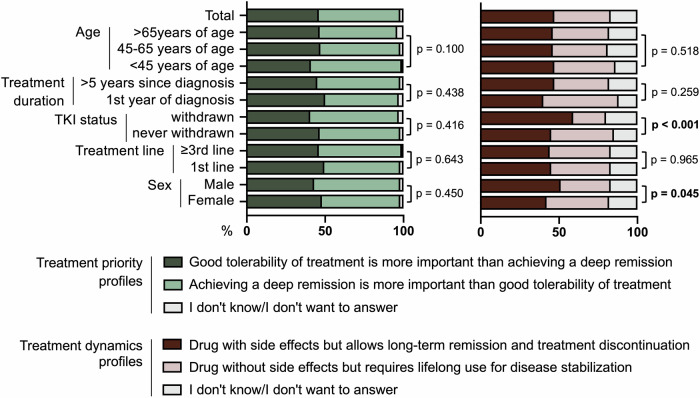
Drug preferences of patients with chronic myeloid leukemia (CML) according to treatment goals. The left panel (“treatment priority profiles”) shows preferences for good tolerability versus achieving a deep molecular remission. The right panel (“treatment dynamics profiles”) shows preferences for a drug with side effects enabling long-term remission and treatment discontinuation versus a well-tolerated drug requiring lifelong intake without side effects. Results are shown for the total cohort and predefined subgroups (age category, time since diagnosis, TKI status, treatment line, and sex). Stacked bars display response distributions; *p* values indicate between-subgroup differences.

Analysis of patient subgroups revealed distinct differences in priorities during TKI therapy. Younger patients ( < 45 years) emphasized fertility (Z = –5.6, *p* < 0.001, r = 0.25), sexuality (–6.5, *p* < 0.001, r = 0.26), and occupational activity (Z = 4.6, p < 0.001, r = 0.19), while older patients ( > 65 years) attached greater importance to mental health (Z = 14.8, *p* < 0.001, r = 0.60) and coping with daily tasks (Z = 10.5, *p* < 0.001, r = 0.43). With increasing disease duration, social interactions and physical activity gained relevance, particularly in long-term survivors (> 5 years) (Z = 7.6, p < 0.001, r = 0.37 and Z = –2.4, *p* = 0.019, r = 0.11, respectively). Treatment history also played a role: patients following TKI discontinuation valued social engagement (Z = 7.1, *p* < 0.001, r = 0.33), sports (Z = 4.8, p = 0.013, r = 0.12), and independence (Z = 15.4, *p* < 0.001, r = 0.73), whereas those without prior discontinuation prioritized occupational functioning (Z = 3.4, *p* < 0.001, r = 0.12,) (Fig. 2). Regarding acceptance of TKI-associated side effects older individuals ( > 65 years) generally displayed greater tolerance toward adverse effects such as nausea (Z = –7.4, *p* < 0.001, r = 0.30), concentration disorders (Z = 7.5, *p* < 0.001, r = 0.31), and skin alterations (Z = 4.0, *p* < 0.001, r = 0.16), whereas younger patients ( < 45 years) rated these toxicities as less acceptable, particularly with regard to reduced libido (Z = 6.4, *p* < 0.001, r = 0.26) and skin changes (Z = 3.99, *p* < 0.001, r = 0.16). Patients in their first year after diagnosis showed comparatively higher acceptance to adverse effects like hair loss (Z = −16.8, *p* < 0.001, r = 0.82) and gastrointestinal symptoms (Z = –9.0, *p* < 0.001, r = 0.44). In contrast, long-term survivors ( > 5 years since diagnosis) were less tolerant of persistent side effects, most notably concentration difficulties (Z = –2.7, *p* = 0.004, r = 0.14) and reduced libido (Z = –7.0, *p* < 0.001, r = 0.34) (Fig. 3). When evaluating therapy-related anxieties, patients reported overall moderate concern that long-term TKI exposure might cause harm to the body (median 3/5), whereas most other worries were less pronounced. Specifically, fears of pain, dying, or not being able to continue therapy, as well as unspecific distress triggered by minor ailments, were reported only occasionally (median 2/5 each). Similarly, fear of medical procedures reached a median of 3, indicating relevance for a subset of patients but not the majority. In contrast to these moderate levels of anxiety, patients’ hopes and expectations were expressed with striking clarity and unanimity: nearly all respondents strongly endorsed the wish to return to normal life, to achieve normal life expectancy under TKI therapy, to prevent cancer recurrence, and ultimately to reach remission with the possibility of treatment discontinuation (all median 5/5) (Supplementary Fig. 1). In the ranking of seven specified treatment goals, ensuring long-term survival emerged as the most consistently prioritized objective across the total cohort, followed by maintaining a largely normal life and preventing relapse (53%, 41%, and 32%, respectively, ranked in the first two positions). Also 28% of patients rated “achieving deep remission“ in the first two positions, whereas the complete absence of side effects and, in particular, treatment discontinuation were less frequently ranked as primary goals (18% and 16%, respectively, ranked in the first two positions) (Fig. 4). Subgroup analyses showed that 23% of patients in the first 12 months and 25% 13 to 24 months after diagnosis ranked “minimizing side effects“ in the first two out of seven positions compared to long-term survivors 25–60 months and more than 60 months after diagnosis (15% and 19%, respectively). Age-related trends further nuanced these preferences: Compared to younger patients (< 45 years) older patients (> 65 years) placed more importance on treatment tolerability and were less focused on prolonging survival (13% vs. 22% and 69% vs. 31%, respectively, ranked in the first two positions) (Fig. 4). In the binary preference scenario 211 (36%) patients favored tolerability and stability, while 271 (47%) patients were prepared to accept adverse effects if this increased the likelihood of achieving deep remission and treatment-free survival (100 (17%) patients abstained) (Fig. 5). In this regard, male patients more often indicated willingness to accept side effects for the chance of discontinuation compared to female patients (51% vs. 42%, χ^2^(2) = 6.81, *p* = 0.045, Cramer´s V = 0.10). Individuals with prior TKI discontinuation more frequently prioritized the possibility of long-term remission, whereas those without such experience tended to favor continuous but well-tolerated therapy (59% vs. 45%, χ^2^(2) = 17.1, *p* < 0.001, Cramer´s V = 0.19). By contrast, preferences were largely unaffected by age, disease duration, or treatment line. The question of whether patients consider treatment tolerability or deep molecular remission to be more important showed similarly balanced results, with no significant subgroup differences (Fig. 5).

## Discussion

This nationwide survey of 582 patients with CML in Germany provides insights into patients’ perceptions of TKI therapy, quality of life, and treatment goal prioritization. The findings confirm and expand on existing literature regarding the increasing relevance of patient-centered outcomes in the long-term management of CML. With a median age of 56 years and 65% of respondents having lived with CML for more than five years, this cohort represents long-term survivors under TKI therapy, although the lower median age compared to the national median at diagnosis (66 years) indicates a selection toward younger patients, likely influenced by the online recruitment process. While overall tolerability of TKI therapy was rated positively (median 4/5), subgroup analyses demonstrated substantial heterogeneity in the perception and acceptance of adverse treatment-related effects. Younger patients prioritized the preservation of fertility, sexual health, and occupational activity, indicating the importance of maintaining social and professional participation early in the course of disease. By contrast, older patients ( > 65 years) designated greater relevance to mental health, independence in daily tasks, and tolerability of therapy, aligning with previously reported age-related shifts in quality of life priorities [23, 24].

Anxieties about future treatment were overall moderate, with concerns about long-term drug-related harm being most pronounced (median 3/5), while fears of dying, therapy failure, or medical procedures were less common. This moderate but persistent level of concern coexisted with consistently high expectations, as most patients strongly endorsed hopes for normal life expectancy, durable disease control, and the possibility of TFR. This coexistence highlights the dual perspective of patients who balance awareness of risks with strong forward-looking aspirations for functional recovery and autonomy. Similar patterns have been described in studies on the psychological well-being of CML patients receiving lifelong targeted therapies, where moderate levels of anxiety coexisted with positive expectations. The studies found that perceived social support played a crucial role in mitigating distress and sustaining long-term resilience [25].

The most highly prioritized treatment goals across the cohort, namely long-term survival, prevention of relapse, and maintenance of quality of life, are consistent with previous surveys [18, 26]. Of note, the recently presented results of the international CML SUN Survey showed that in general doctors place greater emphasis on treatment efficacy, while patients prioritize quality of life and tolerability [27]. Disease control remained the dominant focus, whereas treatment discontinuation and complete freedom from side effects were generally considered less important. These results confirm findings from the survey of Sharf et al., in which patients emphasized disease stability—particularly among those with long-term remission [18]. Nevertheless, subgroup analyses revealed more nuanced preferences: Younger patients (< 45 years) and those with shorter disease duration placed higher value on minimizing side effects, maintaining fertility, occupational planning, and the possibility of pausing therapy. These findings are consistent with results of the LAST study, where it is explicitly emphasized that fertility issues and freedom of treatment are particularly important for younger patients [24]. In contrast, older patients and those with longer treatment duration ( > 5 years) shifted their priorities toward treatment tolerability, psychosocial well-being, and physical resilience. These findings are in line with the concept of the GIMEMA CML Working Party [28] and with findings from the treatment of elderly patients with CML [29]. Over time, many patients appear to experience a “normalization” of life with CML, placing increasing emphasis on functional well-being over curative intent. Attitudes toward TKI discontinuation were ambivalent. While discontinuation was not a top priority in the overall cohort, it was more highly valued by younger patients and those earlier in the treatment course. This trend is consistent with findings from several trials, where interest in TFR was particularly pronounced among younger patients [30, 31]. However, many patients in this survey preferred the safety and tolerability of ongoing TKI treatment to the uncertainty of discontinuation. This perspective was also reflected in the qualitative study by Sharf et al., which showed that patients with acceptable quality of life under TKI therapy were often hesitant to attempt TFR [18]. When asked to choose between a continuous, well-tolerated therapy and a potentially curative but side-effect-prone option, the cohort was nearly evenly divided. Men and patients with prior discontinuation experience were more willing to accept adverse effects in favor of deeper remission. This finding aligns with the work of Breccia et al. [9] and reinforces the 2025 ELN recommendations [7], which advocate for shared decision-making based on patient preferences. However, the results of the CML SUN study show clear discrepancies between doctors and patients in decision-making, with patient involvement often being limited [27]. Treatment choices should therefore consider not only molecular criteria but also individual goals, age, sex, and lived experiences with therapy. The findings underscore that quality of life and participation in everyday activities are critical dimensions of therapeutic success. This is consistent with the recently established expert consensus definition of treatment intolerance in chronic-phase CML, which explicitly frames intolerance in terms of patient-reported impact on daily functioning rather than isolated adverse event grading [32]. While 60% of patients were employed (39% full-time), a substantial proportion (31%) were already retired—often prematurely. These results closely parallel the findings of the Danish nationwide cohort study by Maksten et al., which demonstrated that many CML patients—especially older individuals, women, and those with longer disease duration—struggled to return to the work market despite successful disease control [33].

This study has several limitations. First, voluntary participation through treating physicians and patient advocacy networks may have introduced self-selection bias, as individuals with higher motivation, health literacy, or engagement are more likely to respond to such surveys. As a result, the perspectives reported here may not fully represent the entire CML population, particularly patients less connected to specialized care or support structures. Second, given the large cohort size, even small subgroup differences may have reached statistical significance. Therefore, interpretation was primarily guided by effect sizes (*r*, Cramer’s *V*) according to Cohen’s thresholds, considering only effects of at least medium magnitude ( ≥ 0.3) as clinically meaningful [22].

Taken together, the results of this survey highlight that CML survivorship is characterized by dynamic quality of life priorities that evolve with age, disease trajectory, and prior treatment experience. While survival and disease control remain universal goals, younger patients focus on fertility, work, and future planning, whereas older and long-term survivors prioritize tolerability, independence, and functional well-being. These nuanced perspectives emphasize the need for individualized treatment strategies and shared decision-making that not only consider molecular milestones but also integrate patient-reported quality of life dimensions.

The report on this survey received a poster prize at the European School of Hematology/International CML Foundation 27th John Goldman Conference on Chronic Myeloid Leukemia: Biology and Therapy, October 10-12, 2025, Estoril, Portugal.

## Supplementary information

Supplemental Material

## Data Availability

The datasets are available from the corresponding author on reasonable request.
