# Supplementary material for: Treatment expectations and goals among patients with chronic myeloid leukemia in Germany: a patient-centered perspective
Source: Leukemia. 2025 Dec 19;40(1):29–36. doi: 10.1038/s41375-025-02826-w (PMC12789025; doi:10.1038/s41375-025-02826-w)
Supplement: Supplementary file 1 — Supplemental Material [file 41375_2025_2826_MOESM1_ESM.pdf]

Supplemental Material

Treatment Expectations and Goals among Patients with Chronic Myeloid Leukemia in Germany: A Patient-Centered Perspective

Philipp Ernst[1], Cera Lohse[1,2], Michael Lauseker[3], Jan Geißler[4], Philipp Le Coutre[5], Tim H. Brümmendorf[6], Susanne Saußele[7], Andreas Burchert[8], Georg-Nikolaus Franke[9], Paul La Rosée[10], Annamaria Brioli[11], Thomas Schenk[1], Christian Fabisch[1], Thomas Ernst[1], Guido Mehlkop[2], Andreas Hochhaus[1] on behalf of the German CML Alliance

[1] Klinik für Innere Medizin II, Universitätsklinikum Jena, Comprehensive Cancer Center Central Germany, Campus Jena, Jena, Germany

[2] Institute for Planetary Health Behaviour, Universität Erfurt, Erfurt, Germany

[3] Institut für Medizinische Informationsverarbeitung, Biometrie und Epidemiologie (IBE), Ludwig-Maximilians-Universität, München, Germany

[4] LeukaNET e.V., Riemerling, Germany

[5] Klinik für Hämatologie, Onkologie und Tumorimmunologie, Charité – Universitätsmedizin Berlin, Berlin, Germany

[6] Klinik für Onkologie, Hämatologie und Stammzelltransplantation, Universitätsklinikum RWTH Aachen, Center for Integrated Oncology (CIO) Aachen Bonn Cologne Düsseldorf (CIO ABCD), Aachen, Germany

[7] III. Medizinische Klinik, Universitätsmedizin Mannheim, Universität Heidelberg, Mannheim, Germany.

[8] Klinik für Hämatologie, Onkologie und Immunologie, Universitätsklinikum Marburg, Marburg, Germany

[9] Klinik und Poliklinik für Hämatologie, Zelltherapie, Hämostaseologie und Infektiologie, Universitätsklinikum Leipzig, Comprehensive Cancer Center Central Germany, Campus Leipzig, Leipzig, Germany

[10] Klinik für Innere Medizin II, Schwarzwald-Baar Klinikum, Villingen-Schwenningen, Germany

[11] Klinik für Hämatologie, Hämostaseologie, Onkologie und Stammzelltransplantation, Medizinische Hochschule Hannover, Hannover, Germany

Supplemental Table 1. Additional sociodemographic characteristics of patients with chronic myeloid leukemia (CML) who participated in the survey (n = 582).

		Sex		Treatment line			TKI treatment status		
	Total n=582	Female n=284	Male n=298	1st line n=128	2nd line n=233	≥ 3rd line n=221	On TKI (never withdrawn) n=297	On TKI (after withdrawal) n=143	TKI withdrawn n=142
Family status, n (%)									
Single	105 (18)	51 (18)	54 (18)	22 (17)	42 (18)	41 (19)	66 (22)	20 (14)	19 (13)
Relationship (joint)	408 (70)	191 (67)	217 (73)	98 (77)	161 (69)	149 (67)	200 (67)	105 (73)	103 (73)
Relationship (separate)	21 (4)	10 (4)	11 (4)	3 (2)	8 (3)	10 (5)	9 (3)	5 (3)	7 (5)
Divorced	33 (6)	21 (7)	12 (4)	4 (3)	12 (5)	17 (8)	15 (5)	7 (5)	11 (8)
Widowed	15 (3)	11 (4)	4 (1)	1 (1)	10 (4)	4 (2)	7 (2)	6 (4)	2 (1)
Graduation, n (%)									
No qualification	0 (0)	0 (0)	0 (0)	0 (0)	0 (0)	0 (0)	0 (0)	0 (0)	0 (0)
Main school	68 (12)	21 (7)	47 (16)	11 (9)	34 (15)	23 (10)	35 (12)	15 (10)	18 (13)
Secondary school	192 (33)	114 (40)	78 (26)	37 (29)	82 (35)	73 (33)	89 (30)	47 (33)	56 (39)
High school	322 (55)	149 (52)	173 (58)	80 (63)	117 (50)	125 (57)	173 (58)	81 (57)	68 (48)
Educational level, n (%)									
No qualification	16 (3)	12 (4)	4 (1)	5 (4)	7 (3)	4 (2)	10 (3)	4 (3)	2 (0)
Student	6 (1)	5 (2)	1 (0)	1 (1)	4 (2)	1 (0)	3 (1)	2 (1)	1 (1)
Apprenticeship	136 (23)	73 (26)	63 (21)	21 (16)	64 (27)	51 (23)	64 (11)	29 (20)	43 (30)
Vocational school	76 (13)	53 (19)	23 (8)	15 (12)	32 (14)	29 (13)	39 (13)	17 (12)	20 (14)
Vocational academy	116 (20)	43 (15)	73 (24)	30 (23)	43 (18)	43 (19)	62 (21)	24 (17)	30 (21)
Technical college	63 (11)	18 (6)	45 (15)	14 (11)	28 (12)	21 (10)	34 (11)	19 (13)	10 (7)
University	169 (29)	80 (28)	89 (30)	42 (33)	55 (24)	72 (33)	85 (29)	48 (34)	36 (25)
Current workload, n (%)									
Full-time	225 (39)	70 (25)	155 (52)	51 (40)	90 (39)	84 (38)	121 (41)	49 (34)	55 (39)
Part-time	110 (19)	81 (29	29 (10)	27 (21)	40 (17)	43 (19)	59 (29)	21 (15)	30 (21)
Marginal	9 (2)	7 (2)	2 (19)	2 (2)	6 (3)	1 (0)	3 (1)	3 (2)	3 (2)
Retirement	182 (31)	88 (31)	94 (32)	33 (26)	80 (34)	69 (31)	77 (26)	60 (42)	45 (32)
Parental leave	5 (1)	5 (2)	0 (0)	1 (1)	1 (0)	3 (1)	2 (1)	3 (2)	0 (0)
Retraining	3 (1)	3 (1)	0 (0)	1 (1)	1 (0)	1 (0)	2 (1)	1 (1)	0 (0)
Study/internship	11 (2)	7 (2)	4 (1)	3 (2)	4 (2)	4 (2)	10 (3)	1 (1)	0 (0)
Unemployed	8 (1)	7 (2)	1 (0)	0 (0)	3 (1)	5 (2)	4 (1)	3 (2)	1 (1)
Illness	29 (5)	16 (6)	13 (4)	10 (8)	8 (3)	11 (5)	19 (6)	2 (1)	8 (6)
Current employment, n (%)									
Employee	399 (69)	211 (74)	188 (63)	90 (70)	153 (66)	156 (71)	205 (69)	95 (66)	99 (70)
Worker	38 (7)	15 (5)	23 (8)	9 (7)	17 (7)	12 (5)	18 (6)	7 (5)	13 (9)
Civil servant	57 (10)	21 (7)	36 (12)	15 (12)	21 (9)	21 (10)	28 (9)	16 (11)	13 (9)
Farmer	3 (1)	1 (0)	2 (1)	0 (0)	3 (1)	0 (0)	2 (1)	0 (0)	1 (1)
Self-employed with employee	42 (7)	12 (4)	30 (10)	7 (5)	17 (7)	18 (8)	19 (6)	14 (10)	9 (6)
Self-employed without employees	26 (4)	13 (5)	13 (4)	3 (2)	15 (6)	8 (4)	12 (4)	8 (6)	6 (4)
Student/ trainee	14 (2)	8 (3)	6 (2)	4 (3)	6 (3)	4 (2)	12 (4)	2 (1)	0 (0)
Voluntary year	1 (0)	1 (0)	0 (0)	0 (0)	0 (0)	1 (0)	1 (0)	0 (0)	0 (0)
Never employed	2 (0)	2 (1)	0 (0)	0 (0)	1 (0)	1 (0)	0 (0)	1 (1)	1 (1)

Data are stratified by sex, treatment line, and TKI treatment status. Percentages are given in parentheses. TKI, tyrosine kinase inhibitor.

Supplemental Table 2. Patient knowledge and perceptions of CML and TKI therapy across subgroups and frequency of patient-reported impairments caused by adverse effects of TKI therapy during the last month across subgroups (N = 582).

			Sex		Age (years)			Treatment line				Time since diagnosis (month)				TKI treatment status		
		Total n=582	Female n=284	Male n=298	<45 n=135	45-65 n=326	>65 n=212	1st line n=128	2nd line n=233	≥ 3rd line n=221	Allo SCT n=12	1-12 n=40	13-24 n=39	25-60 n=127	>60 n=205	On TKI (never withdrawn) n=297	On TKI (after withdrawal) n=143	TKI withdrawn n=142
**TKIs are generally well tolerated**	Yes, n (%)	414 (71)	193 (68)	221 (74)	102 (76)	227 (70)	85 (70)	98 (77)	164 (70)	152 (69)	4 (33)	25 (63)	22 (56)	87 (69)	280 (74)	204 (69)	110 (77)	100 (79)
	No	27 (5)	18 (6)	9 (3)	5 (4)	18 (6)	4 (3)	1 (1)	11 (5)	15 (7)	0 (0)	0 (0)	4 (10)	4 (3)	19 (5)	11 (4)	6 (4)	10 (7)
	Not sure	141 (24)	73 (26)	68 (23)	28 (21)	81 (25)	32 (26)	29 (23)	58 (25)	54 (24)	8 (67)	15 (38)	13 (33)	36 (28)	78 (21)	82 (28)	27 (19)	32 (23)
**Treatment goal is to minimise complaints during long-term therapy**	Yes, n (%)	470 (81)	230 (81)	240 (81)	104 (77)	260 (80)	106 (88)	99 (77)	193 (83)	178 (81)	9 (75)	31 (78)	26 (67)	97 (76)	316 (84)	235 (79)	122 (85)	113 (80)
	No	30 (5)	16 (6)	14 (5)	12 (9)	17 (5)	1 (1)	6 (5)	8 (3)	16 (7)	0 (0)	1 (3)	3 (8)	5 (4)	21 (6)	13 (4)	7 (5)	10 (7)
	Not sure	82 (14)	38 (13)	44 (15)	19 (14)	49 (15)	14 (12)	23 (18)	32 (14)	27 (12)	3 (25)	8 (20)	10 (26)	25 (20)	40 (11)	49 (16	14 (10)	19 (13)
**Discontinuation of TKI is usually possible**	Yes, n (%)	221 (38)	115 (40)	106 (36)	58 (43)	116 (36)	47 (39)	47 (37)	89 (38)	85 (38)	3 (25)	10 (25)	11 (28)	49 (39)	151 (40)	96 (32)	57 (40)	68 (48)
	No	107 (18)	59 (21)	48 (16)	32 (24)	66 (20)	9 (7)	22 (17)	46 (20)	39 (18)	2 (17)	5 (13)	7 (18)	25 (20)	70 (19)	56 (19)	28 (20)	23 (16)
	Not sure	254 (44)	110 (39)	144 (48)	45 (33)	144 (44)	65 (54)	59 (46)	98 (42)	97 (44)	7 (58)	25 (63)	21 (54)	53 (42)	156 (41)	145 (49)	58 (41)	51 (36)
**Appr. 50% of patients need to resume TKIs after withdrawal**	Yes, n (%)	264 (45)	135 (48)	129 (43)	70 (52)	156 (48)	38 (31)	52 (41)	110 (47)	102 (46)	2 (17)	10 (25)	13 (33)	58 (46)	183 (49)	121 (41)	60 (42)	83 (58)
	No	38 (7)	15 (5)	23 (8)	13 (10)	20 (6)	5 (4)	10 (8)	13 (6)	15 (7)	1 (8)	2 (5)	4 (10)	7 (6)	25 (7)	20 (7)	11 (8)	7 (5)
	Not sure	280 (48)	134 (47)	146 (49)	52 (39)	150 (46)	78 (64)	66 (52)	110 (47)	104 (47)	9 (75)	28 (79)	22 (56)	62 (49)	169 (45)	156 (53)	72 (50)	52 (37)
**Overall survival rate of CML patients is equal to the general population**	Yes, n (%)	453 (78)	232 (82)	221 (74)	114 (84)	248 (76)	91 (75)	100 (78)	180 (77)	173 (78)	8 (67)	28 (70)	30 (77)	100 (79)	295 (78)	231 (78)	109 (76)	113 (80)
	No	11 (2)	3 (1)	8 (3)	3 (2)	5 (2)	3 (2)	1 (1)	9 (4)	1 (0)	0 (0)	0 (0)	1 (3)	2 (2)	8 (2)	7 (2)	1 (1)	3 (2)
	Not sure	118 (20)	49 (17)	69 (23)	18 (13)	73 (22)	27 (22)	27 (21)	44 (19)	47 (21)	4 (33)	12 (30)	8 (21)	25 (20)	74 (20)	59 (20)	33 (23)	26 (18)
**How often were you annoyed by adverse effects of your treatment in the last month?**	Never, n (%)	159 (27)	51 (18)	108 (36)	38 (28)	86 (26)	35 (29)	28 (22)	69 (30)	62 (28)	2 (17)	9 (23)	8 (21)	33 (26)	109 (29)	77 (26)	39 (27)	43 (30)
	Rarely, n	140 (24)	62 (22)	78 (26)	34 (25)	75 (23)	31 (26)	46 (36)	44 (19)	50 (23)	2 (17)	12 (30)	10 (26)	38 (30)	80 (21)	79 (27)	46 (32)	15 (11)
	Sometimes	146 (25)	82 (29)	64 (21)	31 (23)	86 (26)	29 (24)	40 (31)	63 (27)	43 (19)	2 (17)	12 (30)	13 (33)	35 (28)	87 (23)	94 (32)	38 (27)	14 (10)
	Often	56 (10)	38 (13)	18 (6)	13 (10)	33 (10)	10 (8)	9 (7)	25 (11)	22 (10)	1 (8)	5 (13)	5 (13)	14 (11)	32 (8)	36 (12)	11 (8)	9 (6)
	Every time	13 (2)	10 (4)	3 (1)	7 (5)	5 (2)	1 (1)	2 (2)	5 (2)	6 (3)	0 (0)	1 (3)	3 (8)	3 (2)	6 (2)	7 (2)	5 (3)	1 (1)
	N.a.	68 (12)	41 (14)	27 (9)	12 (9)	41 (13)	15 (12)	3 (2)	27 (12)	38 (17)	5 (42)	1 (3)	0 (0)	4 (3)	63 (17)	4 (1)	4 (3)	60 (42)

Data are stratified by sex, age, treatment line, time since diagnosis, and TKI treatment status. Percentages are given in parentheses. TKI, tyrosine kinase inhibitor.

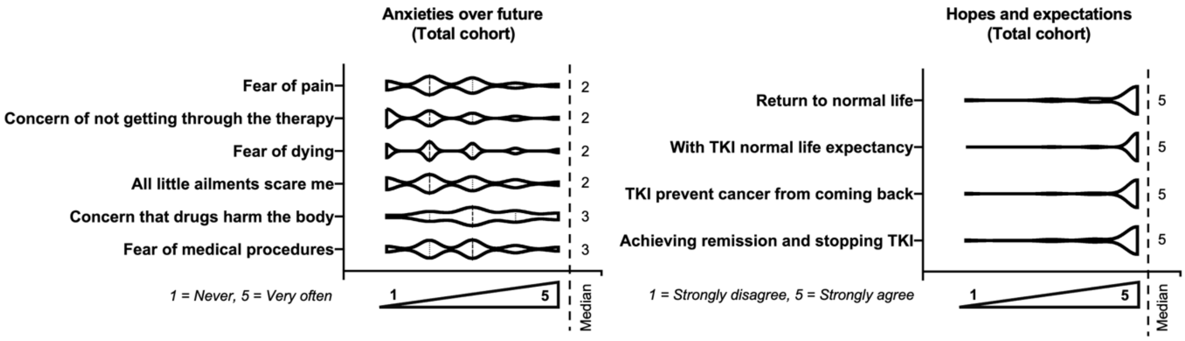

Supplemental Figure 1. Patient-reported anxieties and expectations in chronic myeloid leukemia (CML). Violin plots display responses regarding anxieties about the future (left; 1 = never, 5 = very often) and hopes/expectations of therapy (right; 1 = strongly disagree, 5 = strongly agree). Medians are indicated by dashed lines.
